# Porcelain Teeth

**Published:** 1858-12

**Authors:** Daniel L. Pratt


### PORCELAIN TEETH.

BY DANIEL L. PRATT.

It is a common complaint among dentists, that the various manufacture of porcelain teeth now offered to the profession, are all more or less liable to cracks, chequers, and other injuries, occasioned by the heats which they undergo in the process of soldering to stays and gum plates. From the commencement of my practice in this county, which was in the Fall of 1844, I always doubted the propriety of enveloping and heating porcelain in plaster, and tried various ways, from time to time, in hope of finding some better method. The consequence was, that, on the 3d of November, 1853, I had the good fortune to discover a method that is entirely safe,—neither cracking, checquering, coloring, nor in any way affecting their strength. I have mounted about 2,800 teeth, from four different manufactories, since that period, and have broken but two, in all the various operations; one was broken by a drop of sweat falling on it, when red hot, while in the act of soldering; the other, I can not account for.

My mode of proceeding, is as follows: I grind the teeth to the plates, in the usual way; then envelope them in plaster; then take the teeth out of the envelope, and straighten up the pins with a small beaked forcep, so that the backs be easily relieved from them. I then fit the backs to the teeth, but don't rivet them. I then take wax, prepared from beeswax and gum sandarac (equal parts), warm it till quite soft, then stick the backs to the gum plate as they lay to the teeth, then cut the plaster away, then remove the teeth. I

then take small pointed pins, made of ivory or bone, one-fourth of an inch in length, and pass the points through the pin-holes into the wax, to hold the backs in proper position, while soldering to gum plate; then take plaster, two portions, and brick-dust one portion; lay the work on a porcelain slab, and a ring around it; mix the plaster and brick-dust to the consistence of cream, with water, and pour around it; when the plaster is sufficiently hard, remove the wax, clean the work, and solder; then take off the plaster, clean the work, and rivet the teeth to the backs. I then take Spanish white, three portions, and brick-dust sifted through a piece of bolting-cloth, one portion, mix with 98' alcohol to the consistence of cream; then take a large camel's-hair pencil, and cover the porcelain in every point,—the work will be ready to heat in a few minutes. My reason for employing the brick-dust, is to prevent shrinkage with the plaster and whiting, besides, it retains the heat much longer than either article separate; when the teeth are soldered to the backs, I treat the work in the usual way. I have found work, put up in this way, to be much less liable to breakage, than that which I have heated in plaster. I warrant my work, for an indefinite time, against all fair-play usage; and, of course, if any breakage takes place, it finds its way back to me. The average breakage on work heated in plaster, in my practice, has been one in 35, for a term of five years; while the average in this mode of proceeding, has been only one to 115, for the time I have been working on it,—a period little short of five years.

To prevent brakage in old pieces that have been long worn, when requiring a mend, I take them as they came from the mouth, and cover them up in a cup of sand, put it into a common fire, and let it stand for two hours. The cup should be made of sheet iron, about the size of a common tin cup; the sand should be heated to redness, and let cool before used. Let the work lay about three-fourths of an inch under the surface of the sand; don't let the heat rise above a low red; when taken from the fire, let it stand till cool. It is very rare that a tooth ever cracks, in passing through this process. Mend the work, and coat with the whiting and brick-dust mixed with alcohol, and solder in the usual way; they endure a heat equally well with new teeth.

I do not know whether these methods of treating porcelain are new or not; but it is certain I have not borrowed the idea from any theory, nor received a hint from any man, of a similar character. If they may be of any use to the profession, or any member of it, he is welcome to use them.

